# Dr. Wm. B. Herrick

**Published:** 1846-08

**Authors:** 


### ARTICLE XVII.

Dr. Wm. B. Herrick, one of the editors of this Journal, has received an appointment in the Medical Staff of the U. S. Army, and is at present in Mexico. His labors for the Journal will not, however, cease: he will furnish for its pages a series of letters, embracing everything of interest which may fall under his observation in that country, whether connected directly with his duties, or relating to the medical topography of the places he visits, their advantages for medical men, &c. Should his duties require his absence during the winter, the course of anatomy in the Rush Medical College, will be given as formerly by Dr. Brainard.

